# COVID-19 and Thromboembolic Events in the Pandemic and Pre-Pandemic Era: A Pediatric Cohort

**DOI:** 10.3390/v15071554

**Published:** 2023-07-15

**Authors:** Chiara Rubino, Camilla Bechini, Mariangela Stinco, Donatella Lasagni, Giuseppe Indolfi, Sandra Trapani

**Affiliations:** 1 Pediatric Unit, Meyer Children’s Hospital IRCCS, Viale Pieraccini 24, 50139 Florence, Italy; chiara.rubino@meyer.it (C.R.); camilla.bechini@gmail.com (C.B.); mariangela.stinco@meyer.it (M.S.); donatella.lasagni@meyer.it (D.L.); giuseppe.indolfi@unifi.it (G.I.); 2 Department of NEUROFARBA, University of Florence, Viale Pieraccini 24, 50139 Florence, Italy; 3 Department of Health Sciences, University of Florence, Viale Pieraccini 24, 50139 Florence, Italy

**Keywords:** thrombosis, children, COVID-19, multisystem inflammatory system

## Abstract

The Coronavirus disease 2019 (COVID-19) and multisystem inflammatory syndrome in children (MIS-C) have been variably associated with thromboembolic events (TEs) in children. The aim of our study was to assess the prevalence of TEs in children hospitalized during a five-year period in a tertiary pediatric hospital, particularly in patients with COVID-19 and MIS-C. Overall, 38 patients were discharged with the diagnosis of TE: 20 in the pre-pandemic and 18 in the pandemic period. The prevalence of TEs was the same (0.08%) in the pre-pandemic and pandemic periods. The occurrence of TEs was higher in patients with COVID-19 or MIS-C (6/517, 1.16%) when compared to children without these conditions in the pandemic and in the pre-pandemic periods. The prevalence of TEs in children with MIS-C was significantly higher than the prevalence in patients with COVID-19. Five out of six of the patients with COVID-19 or MIS-C developing a TE had at least one predisposing factor to thrombosis. In conclusion, our study shows an increased prevalence of TEs in children hospitalized with COVID-19 or MIS-C, if compared to children without COVID-19 or MIS-C in the pandemic period and in the pre-pandemic period. The prevalence of TEs was significantly higher in patients with MIS-C.

## 1. Introduction

Thromboembolic events (TEs), rarely described in children, have been increasingly recognized as having a significant impact on mortality and morbidity even in pediatric patients [1,2]. In recent years, their recognition and diagnosis, particularly of venous TEs, have risen due to the greater availability of imaging techniques, the higher level of suspicion, the longer survival of children with chronic diseases that increase the risk of thrombosis, and the more frequent use of medical devices such as central venous line (CVL) [3]. 

Since 2020, the coronavirus disease 2019 (COVID-19), caused by severe acute respiratory syndrome coronavirus 2 (SARS-CoV-2), and multisystem inflammatory syndrome in children (MIS-C) have been variably associated with TEs in children, although definitive data about the thrombotic risk are missing [4,5]. The overall prevalence of thrombosis in children with COVID-19 is much less when compared to adults. This has made it difficult to understand the disease in childhood.

TEs in patients affected by COVID-19 or MIS-C occur more frequently in the venous districts, such as deep veins, central venous sinuses, or pulmonary arteries, but also at a lower rate in the arterial district, such as cerebral arteries, causing acute ischemic stroke, and even more rarely, in the coronaries or peripheral systemic arteries. Intracardiac thrombosis has also been occasionally reported [4]. In our previous comprehensive review, we assessed the literature data concerning 138 children with TEs, with an overall mortality of 12%; notably, analyzing the different sites of TEs, it was slightly higher in patients with arterial TEs (15%) compared to those with venous TEs (11%) [4].

Therefore, the occurrence of TEs represents a challenge for pediatricians as it is a fearsome life-threatening complication, particularly in children with COVID-19 or MIS-C. Several studies tried to define its prevalence with different results.

The aim of this study was to assess the prevalence of TEs in children hospitalized during a five-year period, in a tertiary pediatric hospital, comparing its prevalence in the pre-pandemic and pandemic periods, and in patients hospitalized with and without COVID-19 or MIS-C. In addition, the main demographic, clinical features, and the management of our cohort of patients with COVID-19 or MIS-C developing TEs were described. 

## 2. Materials and Methods

We retrospectively reviewed the medical records of children under the age of 18 years, hospitalized for TEs between 1 May 2017 and 31 December 2022 at Meyer Children’s Hospital IRCCS in Florence, Italy. All the included data were obtained as part of routine clinical activity and were evaluated anonymously. Therefore, specific approval by the ethical committee was not required.

In Italy, hospital discharges are coded following the International Classification of Diseases, 9th revision (ICD-9). For this research, we included codes for thrombophlebitis (451.9), thrombosis (453.9), cerebrovascular event (436), pulmonary embolism (PE) (415.19), gas embolism (9581), venous embolism or deep venous thrombosis of unspecified veins of lower limbs (45.340), deep venous thrombophlebitis of upper limbs (45.183), phlebitis and thrombophlebitis of superficial/deep femoral vein (45.111), venous embolism and deep vein thrombosis of proximal lower limbs (45.341), arterial thrombosis of upper (44.421) and lower limbs (44.422), retinal vascular occlusion (3623), acute myocardial infarction (410), mesenteric ischemia/infarction (5570), and TE of other unspecified veins (4538). 

TE diagnoses were based on different imaging techniques depending on the type of thrombosis: ultrasound for superficial and deep venous thrombosis of lower and upper limbs, ultrasound and/or computed tomography for portal thrombosis, computed tomography for PE, and magnetic resonance imaging for ischemic stroke. 

Our study period has been further divided into two intervals: the pre-pandemic interval, from 1 May 2017 to 28 February 2020, and the pandemic interval, from 1 March 2020 to 31 December 2022. Based on their hospitalization period, also patients with TEs were divided into two groups: Group A, hospitalized in the pre-pandemic interval, and Group B, hospitalized during the pandemic. The association with COVID-19 or MIS-C was assessed for group B. We retrospectively collected data from the patients discharged from Meyer Children’s Hospital, IRCCS with ICD-9 codes for personal history of COVID-19 (V1204), SARS-CoV-2 disease (043), SARS-CoV-2 pneumonia (480.4), and MIS-C (99.590), in the pandemic period. COVID-19 diagnosis was defined in the presence of a positive real-time polymerase chain reaction or antigen SARS-CoV-2 tests on respiratory swabs. MIS-C was defined according to the United States Center for Disease Control and Prevention in the presence of fever, laboratory evidence of inflammation, and clinically severe illness requiring hospitalization, with multisystem (>2) organ involvement; associated with current or recent SARS-CoV-2 infection or exposure to a suspected/confirmed case of COVID-19; in the absence of other alternative plausible diagnoses [6].

TEs associated with COVID-19 or MIS-C were defined as occurring up to three weeks after being diagnosed COVID-19 or MIS-C, as already stated in previous studies [7,8]. 

The clinical and demographic features (age, sex, comorbidity, thrombotic risk factors, and TE site) of patients with TE and COVID-19 or MIS-C, as well as their management, were analyzed. All patient information was accessed through the electronic medical record system. Comorbidity was defined as the occurrence of pre-existing medical conditions. Thrombotic risk factors are conditions known to increase the risk of thrombosis outside the context of SARS-CoV2 infection: active malignancy, systemic infection, burns, venous line, decreased mobility, inflammatory disease, intensive care unit admission, mechanical ventilation, medications (i.e., asparaginase and anticontraceptive drugs), nephrotic syndrome, obesity, recent surgery, severe dehydration, thrombophilia, personal or family history of thrombosis, and cardiopathy [9]. 

Results were summarized as medians and interquartile ranges (IQR) for continuous variables and percentages for nominal variables. Chi-square, Fisher's exact test, and relative risk (RR) were performed to evaluate differences between categorical variables. The value of *p* <  0.05 was considered to be statistically significant, and 95% confidence intervals (CI) are reported. 

Statistical analysis was performed using SPSS (Version 26.0, SPSS, Inc., Chicago, IL, USA) and the freely available “openepi” package (https://www.openepi.com/Menu/OE_Menu.htm, accessed on 31 March 2023).

## 3. Results

In the study period, 38 patients were discharged with the diagnosis of TE: 20 were hospitalized in the pre-pandemic interval (group A) and 18 in the pandemic interval (group B). Overall, the discharged patients in our study period were 46,963 (25,205 in the pre-pandemic interval and 21,758 in the pandemic one). Therefore, children with TEs corresponded overall to 0.08% of the hospitalized patients. The prevalence of TEs was the same (0.08%), both in the pre-pandemic and in the pandemic period. These data are summarized in Table 1. 

Table 2 summarizes a further analysis focused on the results regarding exclusively the pandemic period. Overall, in the pandemic period, 517 children were discharged with the diagnosis of COVID-19 or MIS-C (461 and 56 patients, respectively), and 21,241 patients were not diagnosed with COVID-19 or MIS-C. Notably, one of the 56 patients with MIS-C also had a positive SARS-CoV-2 antigen test at admission, which rapidly became negative within the following 72 h. Since MIS-C was the reason for hospitalization, the patient was included in the MIS-C group. TEs occurred in 12/21,241 children (0.06%) without COVID-19 or MIS-C and in 6/517 (1.16%) children with COVID-19 or MIS-C, notably in 3/461 (0.65%) children with COVID-19 and 3/56 (5.36%) children with MIS-C.

The occurrence of TEs was higher in patients with COVID-19 or MIS-C when compared to children without COVID-19 or MIS-C in the pandemic period (*p <* 0.001, RR 20.54, 95% CI: 7.74–54.52) and to children hospitalized in the pre-pandemic period (*p <* 0.001, RR 14.62, 95% CI: 5.9–36.27).

The prevalence of TEs in the subgroup of children with COVID-19 was significantly higher when compared to patients in the pre-pandemic period (*p <* 0.001, RR 8.2, 95% CI 2.44–27.5) and patients in the pandemic period without COVID-19 or MIS-C (*p <* 0.001, RR 11.52, 95% CI 3.26–40.68). 

The highest prevalence of TEs was observed in the subgroup of children with MIS-C (5.36%); this prevalence was significantly higher compared to all the cases in the pre-pandemic period (*p <* 0.001, RR 67.51, 95% CI 20.64–220.78) and to patients without COVID-19 or MIS-C during the pandemic (*p <* 0.001, RR 94.82, 95% CI 27.5–326.93). The prevalence of TEs in children with MIS-C was significantly higher if compared to patients with COVID-19 (*p*: 0.001, RR 8.23, 95% CI 1.7–39.81). 

The main features of the six children with COVID-19 or MIS-C who developed TEs are summarized in Table 3. Five out of six patients (83.3%) required a period of hospitalization in the Pediatric Intensive Care Unit. The median age was 7 years (IQR: 3.7–8.7, range 1 month–12 years) and 4/6 (66.7%) children were male. Three children (50%) had COVID-19 and three (50%) were diagnosed with MIS-C. Only one patient (1/6, 16.7%) had a comorbidity (medium-chain acyl-CoA dehydrogenase deficiency, and drained bilioma), while the others were previously healthy. Five children (5/6, 83.3%) had a thrombotic risk factor: CVL in three, peripheral venous catheter in one, anti-phospholipid syndrome in one, and previous umbilical catheter in one. Two patients (#2 and #4) had two risk factors: previous insertion of an umbilical venous catheter and obesity, respectively, besides having CVL.

The most frequent TE site was PE in two cases, followed by deep venous thrombosis, portal thrombosis, central retinal artery thrombosis, superficial thrombosis, and central venous sinus thrombosis, each occurring in one patient. Notably, patient #1 developed deep venous thrombosis followed by PE. Only one patient had arterial thrombosis, affecting the central retinal artery and leading to acute blindness; noticeably, this young girl did not have any pro-thrombotic risk factor. No patient had intracardiac TEs on repeated echocardiograms.

None of the patients had received thromboprophylaxis before developing TE. All the children were treated with low-molecular-weight heparin (LMWH); anti-platelet treatment with aspirin was added in the case of the 6-year-old girl with central retinal artery thrombosis. All our patients had a prolonged hospitalization with a median length of stay of 28 days (ranging from 21 to 40 days).

## 4. Discussion

TEs are not common in childhood; therefore, the knowledge of diagnostic, therapeutic, and prophylactic measures is limited among pediatricians. However, their incidence has been increasing in recent years, due to the availability of imaging techniques, the increased survival of medically complex children (suffering from malignancy, prematurity, or surgically corrected defects), and the increasing use of medical devices [10]. A large multicenter retrospective study demonstrated a 70% increase in the annual rate of venous TE, from 35 to 58 cases per 10,000 hospital admissions, in the United States in a 7-year study period (2001–2007) [1]. In the reported cohort, 63% of children had at least one coexisting comorbidity. It is well-known that TEs in children have an age-specific distribution, revealing two peaks, one in the perinatal/neonatal period, and the other in the post-pubertal period [10]. The relatively higher incidence in neonates compared to older children may be due to their higher hematocrit and greater lability of the hemostatic system due to the generally decreased levels of both coagulation factors and their inhibitors in this age group, except factor VIII and the von Willebrand factor which are normal, or even elevated. In adolescents, the incidence equals that of young adults, probably due to hormonal status, the use of contraceptives, pregnancy in young women, obesity, and smoking [10,11]. A recent prospective study demonstrated a prevalence of 1.35% of venous TEs in infants aged < 6 months discharged from Neonatal Intensive Care Units in the United States, with the main clinical risk factors being CVL, total parenteral nutrition, mechanical ventilation, infection, surgery, and extra-corporeal membrane oxygenation [12]. Given the increased incidence and the consequences on morbidity and mortality of TEs in children, applying age-specific models for risk stratification can be crucial to finding patients at a higher risk for TEs, who can benefit from anti-coagulation prophylactic regimens. A prediction model for venous TEs risk in hospitalized pediatric patients has been recently developed and validated; this model includes 11 variables, including a personal history of thrombosis, the presence of CVL, and the co-existence of cardiologic comorbidity [13]. Since the beginning of the COVID-19 pandemic in 2020, hypercoagulability has been extensively described in the course of SARS-CoV-2 infection in adults as an important cause of morbidity and mortality, with prevalence ranging between 0 and 46% of infected adults [14]. Although to a lesser extent when compared to adults, thrombosis has been associated with COVID-19 and MIS-C in children [15]. The main factor for hypercoagulability is thought to be direct and indirect endothelial damage by viral infection and systematic inflammation, leading to the activation of tissue factor pathways, platelets, and multiple cytokines [5,9]. Through similar mechanisms, MIS-C has also been associated with alterations in the inflammatory cascade and hemostasis [5,9,16]. However, COVID-19 and MIS-C seem to have age-specific effects in children, notably on complement activation, leading to hypercoagulability and a thrombogenic state [4,17]. Although variable, the rate of TEs in pediatric patients with SARS-CoV-2 infection is lower than in adults. The reasons are possibly related to differences in the immunologic response to SARS-CoV-2 infection, a higher concentration of antithrombotic serum factors such as alpha-2-macroglobulin in children, the integrity of vascular endothelium in children, differences in the hemostatic response, and age-related variation in thrombosis risk factors like obesity, cardiovascular disease, smoking, and contraceptives use [18,19]. 

In the present study, the prevalence of TEs was significantly higher in children with COVID-19 or MIS-C, if compared to patients without COVID-19 or MIS-C in the pandemic period and to children hospitalized in the pre-pandemic period. 

The prevalence of TEs in children with COVID-19 or MIS-C in our cohort (1.16%) was similar to those reported by Saleh et al. (1.3%) [20], and by Tehseen et al. (1%) [21], and slightly higher than those reported by Beslow et al., (0.82%) [22], and by Antoon et al. (0.4%) [23]. 

The prevalence of TEs in the subgroup of children with COVID-19 in our study (0.65%) was lower than those reported in multicenter studies from an international cohort (1.3%) [21], from Spain (1.1%) [24], and from the United States (1%) [25]. In contrast, the rate was much higher (26%) in the single-center study by Mitchell et al. [26]. 

The prevalence of TEs in the subgroup of children with MIS-C in our cohort (5.36%) was higher than the prevalence reported by Feldstein et al. (2%) [25] and by Tehseen et al. (1%) [21]. On the other hand, a 6.5% prevalence was described by Whitworth et al., who conducted a large multicenter retrospective cohort study to determine the incidence of TEs in children hospitalized with COVID-19 or MIS-C, and the associated risk factors (cancer, central venous catheter, and age older than 12 years) [27].

Overall, our data confirm the results of previously published reports, showing a higher risk of TEs in children hospitalized with COVID-19 or MIS-C. However, the prevalence of TEs was the same in pre-pandemic and pandemic periods; this could be due to the limited time interval and the relatively low number of patients with TEs. 

Most of the patients in our cohort developing TE during COVID-19 or the MIS-C course had at least one predisposing risk factor to thrombosis, in particular the presence of CVL. This finding agrees with the literature findings. Indeed, the most frequently identified risk factor for thromboembolism in childhood has been reported to be the presence of CVL, which accounts for over 90% of neonatal venous TE and over 50% of pediatric VTE [28]. Additionally, in pediatric health care, CVLs have globally improved the management of severely ill patients, such as those affected by tumors, severe infections, short-bowel syndrome, or prematurity, who require frequent blood tests, chemotherapy, and total parenteral nutrition. Children with COVID-19 and, even more, those with MIS-C, can have a severe course of the disease and might often require the use of these devices.

Therefore, the use of CVL is progressively increasing, with approximately 25% of hospitalized children requiring them [29]. 

Prothrombotic comorbidities have been described in several other cohorts as increasing the risk of TEs in patients with COVID-19 and MIS-C [21,22,24,26,27]. These predisposing conditions overlap those described in literature studies in children with TEs occurring regardless of SARS-CoV-2 infection [2,4,10]. In our cohort, obesity was reported in only a 9-year-old girl who developed PE. Obesity is a well-known independent risk factor of venous TE for hospitalized patients. In addition, obesity is associated with the requirement of respiratory support under intubation and the development of acute respiratory distress syndrome and other respiratory failure [30]. In a recent study by Tehseen et al., on children with MIS-C and TEs, obesity and the use of respiratory support were significantly more common than in those with MIS-C without TEs [21].

Thus, in COVID-19 and MIS-C, obesity may lead to increased risks of the development of thrombosis and the worsening of such conditions in children.

The majority of our patients had TEs in the venous district as already reported in the literature: venous TEs have been reported as the most frequent thrombotic complications, representing more than half of the published cases. Among them, PEs and deep venous thrombosis are the most frequent, sometimes with simultaneous localizations [4]. In our cohort, the arterial localization of TEs was observed only in a 6-year-old girl who developed central retinal artery thrombosis leading to acute blindness; so far, this case is the first COVID-19-related central retinal artery thrombosis in childhood. We did not have any cases of intracardiac thrombosis.

Almost all our patients with such a complication have requested admission to Pediatric Intensive Care Unit and they all needed a very long hospital stay, confirming the severe course of the disease.

The indications and benefits of thromboprophylaxis in children hospitalized for COVID-19 and MIS-C are still debated. Early in the pandemic, consensus guidelines suggested low-dose LMWH for children hospitalized with COVID-19 or MIS-C having additional venous TE risk factors (such as CVL, mechanical ventilation, obesity, malignancy, and cardiac disease) or having D-dimer level at least five times the upper limit of normal, unless there are contraindications to anticoagulation [31]. However, these guidelines were based on experts’ opinions, and they were developed before adult thromboprophylaxis trials and most of the pediatric studies assessing TE incidence and risk factors [5]. Elevated D-dimers were associated with TEs in some pediatric studies but their use as a predictor of thrombosis is limited because of the variation in timing and frequency of testing [4,5]. Therefore, further data are necessary to confirm the above-cited indications for thromboprophylaxis. Single-center studies suggested its use in children with COVID-19 or MIS-C with a tailored approach [8,32]. A phase-2 multicenter study demonstrated the safety of twice-daily enoxaparin (initial dose: 0.5 mg/kg per dose; max: 60 mg; target anti-Xa activity: 0.20–0.49 IU/mL) as primary thromboprophylaxis for children < 18 years of age hospitalized for symptomatic COVID-19, including primary respiratory infection and MIS-C [33]. No relevant bleeding or other serious adverse effects were observed; further investigations are needed to assess clinical efficacy. According to the most recent recommendations on MIS-C by the American College of Rheumatology, anticoagulation should be added to antiplatelet therapy in patients with cardiac involvement and considered “case by case” in other patients, taking into account the presence of risk factors for thrombosis or bleeding [34]. Moreover, the majority of TEs in the multicentric study of Whitworth et al. developed in patients receiving thromboprophylaxis [27]. This has raised questions regarding the optimal intensity of prophylactic anticoagulation for patients with COVID-19 or MIS-C.

The main limitations of our study are its retrospective design and the use of ICD-9 codes to select the patients, which may lead to an underestimation of the cases with TEs. Another limitation is the design of the monocentric type of study; this has implied a relatively low number of patients with TE complications in both the pre-pandemic and pandemic periods. In addition, the short duration of the period of study and, therefore, the low number of patients with TEs may contribute to the absence of differences in TE prevalence between pre-pandemic and pandemic periods. Finally, we could not compare the demographic and clinical features, as well as the use of thromboprophylaxis, in children with and without COVID-19 or MIS-C as these data were unavailable for patients hospitalized in the pre-pandemic period. 

## 5. Conclusions

TEs, although rare, might represent a leading cause of morbidity and mortality in children with COVID-19 and MIS-C. According to the literature, our study confirmed that these complications occur with a higher incidence in this specially selected population rather than in children hospitalized for other reasons. Indeed, we showed an increased prevalence of TEs in patients hospitalized in a tertiary children’s hospital with COVID-19 or MIS-C, if compared to children without COVID-19 or MIS-C in the pandemic period and to children in the pre-pandemic period. The prevalence of TEs was significantly higher in patients with MIS-C. In the pandemic period, most TEs occurred in the presence of at least one risk factor for thrombosis, mostly represented by CVL.

The occurrence of such complications often required Intensive Care Unit admission and a long hospitalization in our cohort, confirming that TEs might have a significant impact on children’s health and overall health costs.

Therefore, pediatricians facing children with COVID-19 or MIS-C should be aware of the true possibility and implications of TEs. 

## Figures and Tables

**Table 1:** Number of patients discharged from Meyer Children’s Hospital IRCCS and those with TEs in the study period, in the pre-pandemic and in the pandemic intervals.

	Total	Pre-Pandemic	Pandemic
Overall	46,963	25,205	21,758
TEs *n (%)*	38 (0.08%)	20 (0.08%)	18 (0.08%)

*n*: number, TEs: thromboembolic events.

**Table 2:** Number of patients with and without COVID-19 or MIS-C diagnosis, and with and without TEs in the pandemic period. TEs were significantly more frequent in patients with COVID-19 or MIS-C if compared to patients without COVID-19 or MIS-C.

	Without COVID-19 or MIS-C	With COVID-19 or MIS-C	COVID-19	MIS-C
**Overall**	21,241	517	461	56
**TEs *n (%)***	12 (0.06%)	6 (1.16%)	3 (0.65%)	3 (5.36%)

COVID-19: Coronavirus disease 2019, MIS-C: Multisystem inflammatory syndrome in children, *n*: number, TEs: thromboembolic events.

**Table 3:** Main clinical feature of patients with COVID-19 or MIS-C who developed TEs.

	Patient 1	Patient 2	Patient 3	Patient 4	Patient 5	Patient 6
Age/sex	8 y/M	1 m/M	6 y/F	9 y/F	12 y/M	3 y/M
Comorbidity	None	Medium-chain acyl-CoA dehydrogenase deficiency, drained bilioma	None	None	None	None
COVID-19/ MIS-C	COVID-19	COVID-19	COVID-19	MIS-C	MIS-C	MIS-C
TE	Deep venous thrombosis (right popliteal vein and small saphenous vein), PE	Portal thrombosis	Central retinal artery thrombosis	PE	Superficial venous thrombosis (cephalic vein)	Central venous sinus thrombosis
Thrombotic risk factors	Anti-phospholipid syndrome	CVL, previous umbilical venous catheter	None	CVL Obesity	PVC in cephalic vein (midline)	CVL
Management	LMWH	LMWH	LMWH, ASA	LMWH	LMWH	LMWH

ASA: acetyl salicylic acid; COVID-19: Coronavirus disease 2019, CVL: central venous line, F: female, LMWH. Low-molecular-weight heparin, M: male, m: month, MIS-C: Multisystem inflammatory syndrome in children, PE: pulmonary embolism, PVC: peripheral venous catheter; TE: thromboembolic event, y: year.

## Data Availability

Data sharing is not applicable to this article.
